# Gastrointestinal Symptoms and Elevation in Liver Enzymes in COVID-19 Infection: A Systematic Review and Meta-Analysis

**DOI:** 10.7759/cureus.9999

**Published:** 2020-08-24

**Authors:** Srinivas Puli, Muhammad Baig, Saqib Walayat

**Affiliations:** 1 Gastroenterology, University of Illinois, Peoria, USA

**Keywords:** covid 19, nausea and vomiting, lft - liver function tests, low albumin

## Abstract

Background

Corona virus has become a global health pandemic since its origin in Wuhan, China, in December 2019. The present systematic review and meta-analysis aims to assess gastrointestinal symptoms and liver enzymes trend in corona virus infection.

Methods

Pooled proportions were calculated using both fixed and random effects model. Weighted mean difference and 95% CI were calculated.

Results

We included 15 relevant articles in the meta-analysis (n = 3762). The pooled proportion of patients with nausea/vomiting was 7.00% (95% CI = 6.00-8.00) while that of diarrhea was 6.00% (95% CI = 5.00-7.00). Weighted mean difference of aspartate aminotransferase (AST), alanine aminotransferase (ALT) in non-severe COVID-19 patients was higher by 20.54 U/liter (95% CI = 19.95-21.13) and 21.38 U/liter (95% CI = 20.92-21.84) while that in severe patients was higher by 22.70 U/liter (95% CI = 19.09-26.45) and by 22.94 U/liter (95% CI = 20.46-25.42) respectively, as compared to general population. Pooled proportions showed ALT levels to be elevated in 16.00% (95% CI = 14.00-18.00) of patients with non-severe and 25.00% (95% CI = 20.00-31.00) of patients with severe COVID-19. Weighted mean difference of albumin and platelet count was found to be lower by 3.28 g/liter (95% CI = 3.05-3.50) and by 14.21 x 10^9^/liter (95% CI = 11.87-16.55) in non-severe patients and by 11.39 g/liter (95% CI = 10.16-12.63) and 40.70 x 10^9^/liter (95% CI = 33.62-47.77) in severe patients.

Conclusions

Our meta-analysis shows that patients with COVID-19 infection can present with nausea, vomiting and diarrhea in addition to elevated liver enzymes including AST, ALT and a decline in albumin and platelet count which is more marked in severe disease.

## Introduction

In December of 2019, the outbreak of the novel coronavirus (SARS-CoV-2) from Wuhan, China changed the world as we knew it. While initially those with exposure to the seafood market in Wuhan were infected, soon individuals throughout the province began experiencing symptoms of COVID-19. By the end of January 2020, the United States (US) experienced their first case of COVID-19, and as of April 1, 2020 the number of cases in the US has surpassed all the other countries in the world.

COVID-19 is a disease caused by SARS-CoV-2, a novel coronavirus related to the virus that caused the SARS (severe acute respiratory distress syndrome) outbreak in 2002 (SARS-CoV) and MERS (Middle East respiratory syndrome) outbreak in 2012. While the fatality of SARS-CoV-2 is lower than that of SARS or MERS, there is a much larger rate of person to person transmission due to its wide spectrum of disease manifestations in individuals.

Like SARS and MERS, COVID-19 is known to have catastrophic complications such as acute respiratory distress syndrome (ARDS), severe pneumonia, acute respiratory failure, and acute cardiac events as reported out of China [1]. Less severe symptomatology includes sore throat, cough, low-grade fevers, and malaise. Currently the Centers for Disease Control and Prevention (CDC) warns the community to look out for symptoms of fever, cough, and sore throat when suspicious for COVID-19.

We are now receiving data out of China and Italy about characteristics of patients with COVID-19. It is being reported that gastrointestinal (GI) symptoms were commonly seen in infected COVID-19 patients [2]. Of note, the first case of COVID-19 (in Seattle, Washington) manifested as cough and fever, followed by nausea, vomiting, and loose stools [3]. The authors of this case reported that the infected individual initially had malaise and gastrointestinal symptoms (nausea, vomiting, abdominal pain, loose stools), and did not have respiratory distress until later in the disease course (day 9).

Currently, literature on COVID-19 continues to remain limited, while it continues to spread all around the world. It remains crucial for us to understand symptoms and various presentations in order to identify the disease early on and help in containment. Moreover, digestive symptoms can also be important as patients with digestive symptoms have been reported to have longer hospital and more severe disease [2].

The objective of this review and metanalysis was to review available literature since onset of disease with special focus on gastrointestinal symptoms and elevated liver enzymes to further help with the early recognition, prevention, and containment of the currently ongoing pandemic.

## Materials and methods

A literature search was conducted using the electronic database engines MEDLINE through PubMed, Ovid, Cochrane Library, EMBASE, Cumulative Index for Nursing & Allied Health Literature, DARE, International Pharmaceutical Abstracts, OVID Health star and Google Scholar from 2019 to March 31st, 2020 to identify published articles using keywords ‘Novel Corona virus’, ‘Coronavirus 2019’, ‘COVID-19’, ‘SARS-CoV-2’, ‘nausea, vomiting’, ‘diarrhea’ or ‘elevated liver enzymes’. These terms were used in various combinations in the above-mentioned data bases. The reference list of all eligible studies was reviewed to identify additional studies. The retrieved studies were carefully examined to exclude potential duplicates or overlapping data. Titles and abstracts selected from the initial search were first scanned, and the full papers of potential eligible studies were reviewed.

Study eligibility

We included peer reviewed studies, case series with reverse transcription-polymerase chain reaction (RT-PCR) confirmed COVID-19 infection. Studies were eligible for inclusion if they reported COVID-19-related GI symptoms including nausea, vomiting, diarrhea, and/or elevated liver enzymes. Articles were excluded if (1) they were not written in English, (2) no outcomes were reported, or (3) they represented review articles or studies published as abstracts only. Two reviewers (SW, MB) independently performed study selection according to eligibility criteria. Disagreements were resolved by discussion with a third reviewer (SP).

Data extraction and quality assessment

The following data were independently abstracted onto a standardized form: study characteristics (primary author, year of publication, and country of the population studied), study design, baseline characteristics of the study population (number of patients included, sex, age of patients, risk stratification if mentioned into mild/moderate or severe disease), and outcomes (nausea, vomiting, diarrhea, elevated aspartate aminotransferase (AST), alanine aminotransferase (ALT), total bilirubin, albumin and platelet count).

Outcomes of interest

We evaluated proportion of patients with symptoms of nausea, vomiting and diarrhea in all COVID-19 positive patients and proportion of patients with nausea vomiting and diarrhea in severe COVID-19 patients. We also assessed the weighted mean difference of AST, ALT, albumin and platelet count in all COVID-19 positive patients and in patients with severe COVID-19 as compared to general population. Severe COVID-19 was defined as severe community acquired pneumonia based on American Thoracic Society (ATS) guidelines, or patients requiring admission to intensive care unit (ICU) due to COVID-19, or respiratory distress, respiratory rate > 30 breaths per minute, oxygen saturation <93% at rest, partial pressure of oxygen/FIO2<300, acute respiratory distress syndrome (ARDS), shock arrhythmia or multiorgan failure. All other cases were classified as non-severe infection.

Statistical analysis

This meta-analysis was performed, with calculation of weighted mean difference and 95% confidence interval (CI). Mean and standard deviations were calculated for continuous variables.

Pooled proportions and 95% CI were used to summarize the weighted effect size for each subgroup proportions. First the individual study proportions were transformed into a quantity using Freeman-Tukey variant of the arcsine square root transformed proportion. The pooled proportion is calculated as the back-transform of the weighted mean of the transformed proportions, using inverse arcsine variance weights for the fixed effects model and DerSimonian-Laird weights for the random effects model [4]. Forrest plots were drawn to show the point estimates in each study in relation to the summary pooled estimate. The width of the point estimates in the Forrest plots indicates the assigned weight to that study. The heterogeneity among studies was tested using I2 statistic and Cochrane Q test based upon inverse variance weights [5]. I2 of 0% to 39% was considered as non-significant heterogeneity, 40% to 75% as moderate heterogeneity, and 76% to 100% as considerable heterogeneity. If P-value is >0.10, it rejects the null hypothesis that the studies are heterogeneous. The effect of publication and selection bias on the summary estimates was tested by both Harbord-Egger bias indicator and Begg-Mazumdar bias indicator. Also, funnel plots were constructed to evaluate potential publication bias [6].

## Results

**Figure 1:**
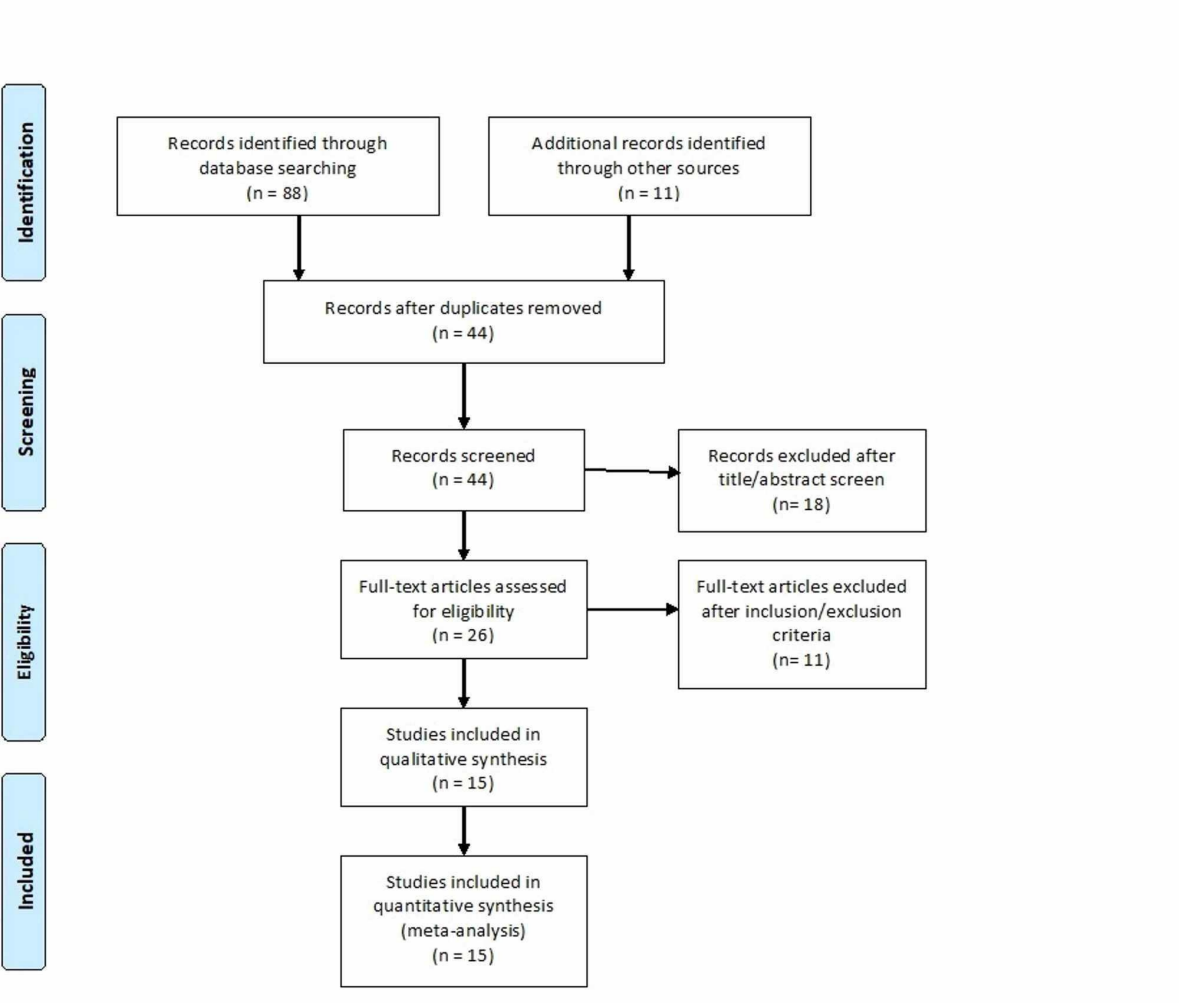
Preferred reporting items for Systematic Reviews and Meta-analyses flow diagram (PRSIMA) for detailing the review process.

Initial search identified 88 articles based on our search criteria. After thorough screening and removal of abstracts, review papers, and duplicates 15 articles were included in our meta-analysis with a total of 3762 cases, 404 patients with severe COVID-19. A preferred reporting items for Systematic Reviews and Meta-analyses flow diagram for detail of the review process is shown in Figure 1. All studies were published as full text articles. All pooled estimates given are estimates calculated by fixed effect model. Fixed effect model was preferred to random effects model for better accuracy based on the nature of individual study characteristics and heterogeneity.

Among the included studies, 13 were based in China, one in USA, and one in France. Mean age of all COVID-19 patients was 48 years, while that of patients afflicted with severe COVID-19 was 60.8 years. The P for Chi-squared heterogeneity for all the pooled accuracy estimates was >0.10. The agreement between reviewers for the collected data gave a Cohen κ value of 1.0.

Outcomes of interest

Nausea/Vomiting/Diarrhea

**Figure 2:**
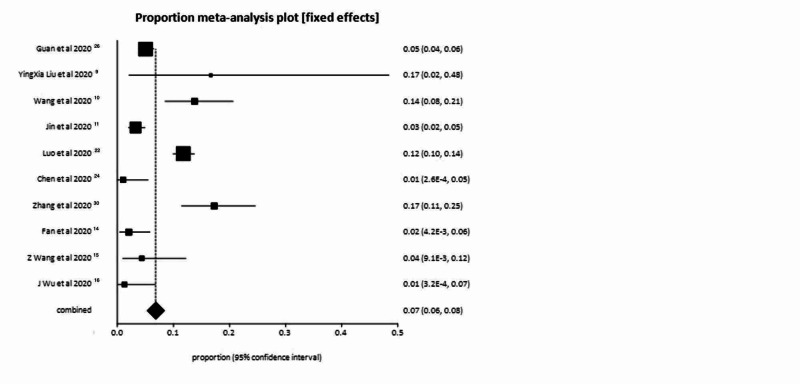
Forrest plot representing individual study proportions and the pooled estimates of nausea and vomiting.

**Figure 3:**
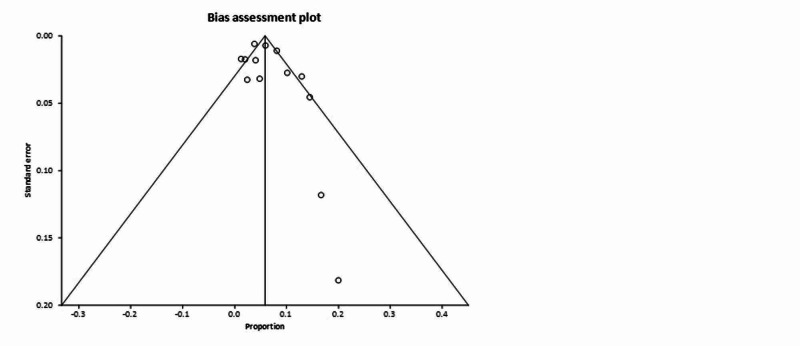
Funnel plot assessing the publication bias for diarrhea.

In the pooled analysis, 7.00% of patients had symptoms of nausea or vomiting or both (95% CI = 6.00-8.00). Publication bias calculated using Begg-Mazumdar indicator gave Kendall’s tau b value of 0.29 (P > 0.05). Forrest plot representing pooled and individual results of studies with nausea and vomiting is shown in Figure 2. The same publication bias calculated using Harbord-Egger bias indicator gave a value of 1.24 (95% CI = -3.28 to 5.76, P = 0.54). Both indicators showed that there was no publication bias. Funnel plot assessing the publication bias is shown in Figure 3. In our pooled analysis, percentage of patient who had diarrhea was 6.00% (95% CI = 5.00-7.00).

Elevation in AST/ALT and total bilirubin in COVID-19 patients

**Figure 4:**
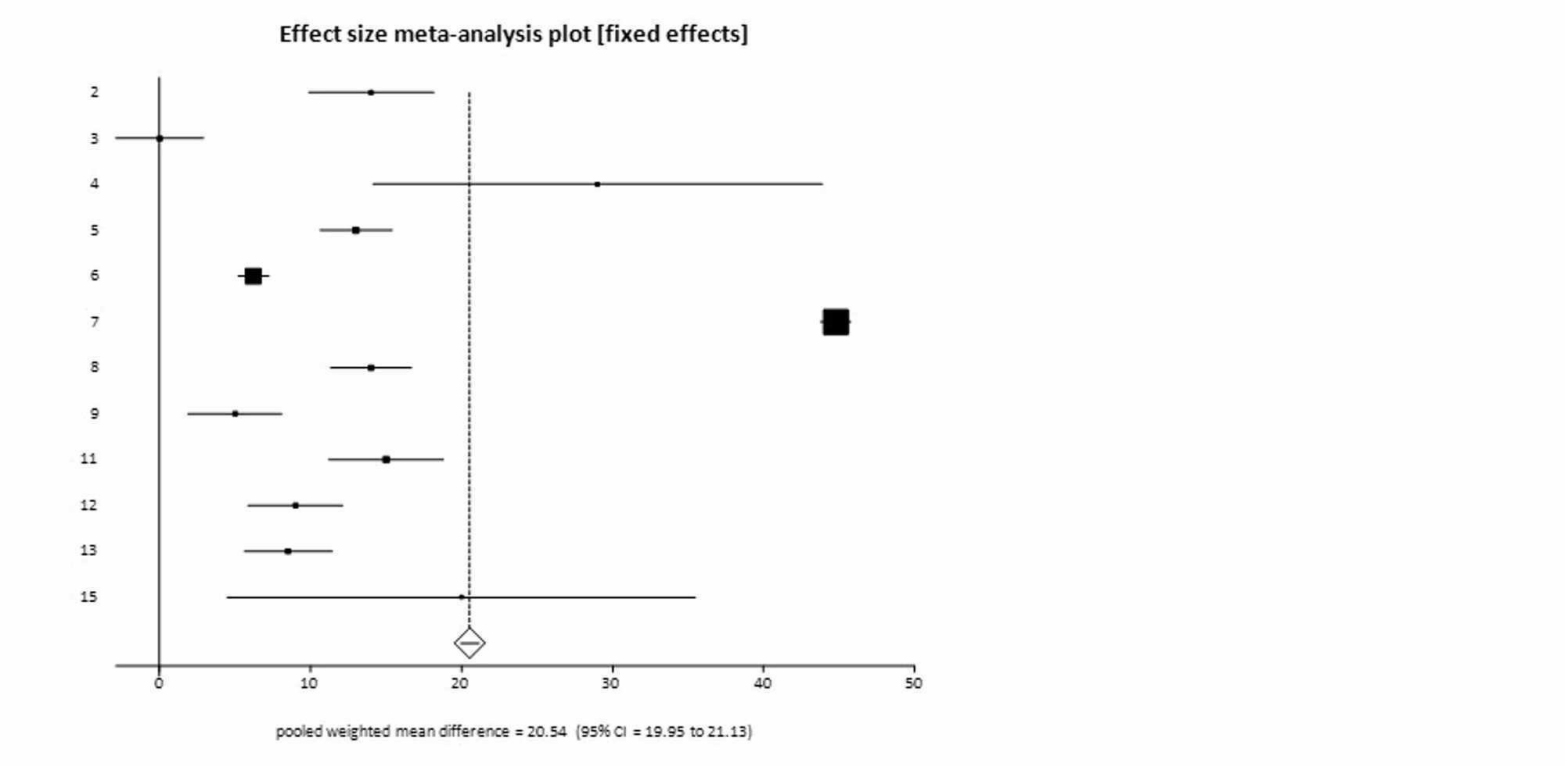
Forrest plot representing individual and pooled estimates of studies with elevated aspartate aminotransferase (AST).

The weighted mean difference of AST levels in all COVID-19 patients was higher by 20.54 U/liter (95% CI = 19.95-21.13) as compared to general population. Forrest plot representing pooled and individual results of studies with AST levels is mentioned in Figure 4. The weighted mean difference of AST in severe patient compared to general population was higher by 22.77 U/liter (95% CI = 19.09-26.45).

As compared to general population, the weighted mean difference of ALT was higher in all COVID-19 patients by 21.38 U/liter (95% CI = 20.92-21.84). Weighted mean difference of ALT in severe patients as compared to general population was higher by 22.94 U/liter (95% CI = 20.46-25.42). Pooled analysis showed, ALT levels were elevated in 16.00% (95% CI = 14.00-18.00) of patients with non-severe COVID-19 and in up to 25.00% (95% CI = 20.00-31.00) of patients with severe COVID-19. Pooled proportion showed that total bilirubin was found to be elevated in 7% (95% CI = 6.00-9.00) of COVID-19 positive patients.

Mean albumin in COVID-19 patients

Weighted mean of albumin was found to be lower by 3.28 g/liter (95% CI = 3.05-3.50) in patients with COVID-19 as compared to general population. In severe COVID-19 patients the weighted mean of albumin was lowered by 11.39 g/liter (95% CI = 10.16-12.63) as compared to general population.

Mean platelet count in COVID-19 patients

The weighted mean platelet count in COVID-19 patients was found to be lower by 14.21 x 10^9^/liter (95% CI = 11.87 to 16.55) as compared to general population. While in severe patients, the weighted mean platelet counts further downtrended by 40.70 x 10^9^/liter (95% CI = 33.62-47.77) as compared to general population.

## Discussion

COVID-19 is an emerging, exponentially spreading viral illness which has engulfed almost the entire human population since its onset about four months ago. Since its origin from Wuhan, China, it has dramatically affected the landscape of our social and medical communities. Our ability to combat this pandemic is limited by our finite understanding of this novel virus. While pulmonary symptoms are reported in more than 90% of patient population more data seems to be emerging about gastrointestinal symptoms [2,7,8]. In this meta-analysis we analyzed and evaluated gastrointestinal symptoms and laboratory characteristics of more than 3500 patients in order to further improve our understanding and to mitigate risks [9-16].

We only assessed nausea, vomiting and diarrhea as they were commonly reported symptoms among the studies included in our pooled analysis. Nausea/vomiting or both were reported in about 7% of our population cohort presenting with COVID-19 infection. These results are much higher than Li et al. who reported nausea and vomiting to be present among 3.9% of their population cohort [17]. Our results are significant since our patient cohort was larger as compared to theirs (1994 patients). These rates are much lower than Middle east respiratory distress syndrome (MERs-CoV) in which nausea and vomiting was reported in up to 21% of patients [18].

Diarrhea was reported in up to 6% of patients in our population cohort. Earlier studies have reported rates of up to 4.8%-6% [17,19]. Diarrhea was reported in up to 25% of patients in earlier MERS-CoV and SARS [20]. Characteristics of COVID-19-related diarrhea remain poorly defined; Pan et al. mentioned diarrhea up to three times daily [2]. Duration of diarrhea remains unclear currently; previously in SARS-CoV self-limiting diarrhea was reported for up to four days [21]. Further studies will likely help in further delineating characteristics of COVID-19-related diarrhea.

Other GI symptoms that have been reported included abdominal pain, and anorexia which is non-specific. Luo et al. reported GI symptoms in 16% of their patient population of 1181 patients [22]. Most common GI symptom interestingly in their population cohort was loss of appetite in 98% of patients, followed by nausea in 73%, vomiting in 65%, diarrhea in 37%, and abdominal pain in 25% of patients [22]. Anorexia has been reported in 83% of their cases by Pan et al. (83/99), and abdominal pain was reported only in four cases out of 99 in their cohort [2].

The etiology of GI manifestations in COVID-19 is thought to be related to viral invasion of angiotensin converting enzyme II expressing cells that can be found in higher quantities in upper esophagus, glandular cells of stomach, duodenum, absorptive enterocytes in ileum and colonic mucosa [21-23]. These cells serve as the likely entrance to GI tract for corona virus as ACE-II receptor is required for viral entrance into cells. Subsequently, following viral entrance an inflammatory cascade ensues along with enteric nervous system activation leading to cell destruction, viral shedding, and GI-related symptoms [23]. Xiao et al. showed viral shedding to be present in >50% of their patients. This persisted for up to 12 days in some cases and continued even after clearance of virus from respiratory tract [21]. Other studies have also reported continued viral shedding even after negative nasopharyngeal testing. This supports feco-oral transmission of the virus and is of utmost importance as patients may continue to spread infection post recovery when they are asymptomatic [2,24].

Underlying chronic liver disease (Chronic hepatitis B, fatty liver or unspecified chronic liver disease) was reported in 3% of our patient population (70/2279). Our data is in accordance with previous studies which report chronic liver disease in 2-11% of patients with COVID-19 [25]. Data regarding liver enzymes is conflicting. While some previous case series have suggested that liver enzymes elevation is present in up to 50% of patients with more marked elevations in severe COVID-19 cases [25, 26]. Other studies have reported no significant elevations in liver enzymes [2,27]. In our pooled analysis, liver enzymes were reported to be elevated in 16.00% of patients with non-severe COVID-19 and in up to 25.00% of patients with severe COVID-19. However, our weighted mean difference of AST in non-severe and severe disease did not show any remarkable difference. While weight mean difference of AST in non-severe COVID-19 patients was 20.54 U/liter, that in severe COVID-19 patients was also almost similarly elevated as compared to general population 22.77 U/liter. Elevations in ALT were also almost similar to that of AST, with weighted mean difference of ALT in non-severe COVID-19 patients being higher by 21.38 U/liter and that in severe COVID-19 patients being higher by 22.94 U/liter as compared to general population. These results indicate that while liver enzymes could be elevated in patients with COVID-19 infection, there is likely not enough significant difference of elevation in between severe and non-severe infection. To date there have been no cases of COVID-19-related liver failure reported to our knowledge.

The etiology of hepatocellular injury is thought to be multifactorial and could be related to direct viral injury, drug-induced liver injury and ischemic hepatopathy in the setting of ARDS and multiorgan failure. This is supported by the fact liver enzymes uptrend with disease progression [25]. Previous studies have shown viral predilection for bile duct cells as they express higher quantities of ACE-II as compared to hepatocytes which express ACE-II only minimally. These bile duct cells upregulate ACE-II in response to liver injury leading to proliferation of hepatocytes which could be the possible mechanism of liver injury in patients with COVID-19 [28]. Total bilirubin was elevated in 7% of COVID-19 positive patients in our cohort. We did not elevate alkaline phosphatase or gamma glutamyl transferase (GGT) because of limited number of studies reporting these parameters; GGT was reported to be elevated in 50% of patients previously [25]. Pathological evidence from autopsy showed lack of viral inclusion bodies on autopsy [28]. SARS-CoV has been reported to cause liver injury in 60% of patients previously and has also been isolated from liver tissues on biopsy [21]. Further studies need to be done in order to further evaluate laboratory patterns, clinical and pathological changes in liver injury in COVID-19 infection.

Our population cohort was also noticed to have thrombocytopenia and hypoalbuminemia which was more marked in patients with severe disease. This is in accordance with previous studies [19]. In our study weighted mean difference of platelet count was found to be lower by almost 40.70 x 10^9^/liter in severe COVID-19 infection. A previous meta-analysis had reported weighted mean difference of platelet count to be lower by 31 x 10^9^/liter in severe COVID-19 patients [29]. The etiology of thrombocytopenia is likely multifactorial in the setting of infection including consumptive coagulopathy, disseminated intravascular coagulation (DIC) and is likely a marker of severe disease and organ dysfunction along with albumin [29, 30]. Hypoalbuminemia has been reported in 50% of patients previously with non-severe disease and in 90% of patients with severe disease. Hypoalbuminemia is likely related to decreased synthesis, increased catabolism, and increased vascular permeability in the setting of sepsis and shock [30].

Our meta-analysis had several limitations. Most of our studies were retrospective case series, definition of severe disease differed among various studies, different cut offs for AST, ALT, total bilirubin, albumin and platelet counts may limit interpretation of analysis. Most of the studies included were from China and future studies from USA and European disease pattern may help to further characterize the disease.

## Conclusions

Clinicians must be aware of gastrointestinal symptoms of COVID-19 infection. Patients may present solely with GI symptoms without pulmonary involvement which should be recognized early on to prevent rapid transmission. More research needs to be done in order to further characterize incidence, prevalence and significance of gastrointestinal symptoms and elevation in liver enzymes in COVID-19 infection. In the meanwhile, in patients presenting with nausea, vomiting and diarrhea COVID-19 should be kept on the differentials as this may lead to early identification, isolation and decrease health care burden. Further studies are also needed to assess implications of stool testing and role of endoscopic precautions in the setting of asymptomatic viral shedding in stool.
